# Global Origin of *Mycobacterium tuberculosis* in the Midlands, UK

**DOI:** 10.3201/eid1603.090813

**Published:** 2010-03

**Authors:** Jason T. Evans, Sarah Gardiner, E. Grace Smith, Richard Webber, Peter M. Hawkey

**Affiliations:** Health Protection Agency West Midlands Laboratory, Birmingham, UK (J.T. Evans, S. Gardiner, E.G. Smith, P.M. Hawkey); King’s College London, London, UK (R. Webber); University of Birmingham, Birmingham (P.M. Hawkey)

**Keywords:** Molecular epidemiology, Mycobacterium tuberculosis, geography, humans, tuberculosis and other mycobacteria, bacteria, United Kingdom, dispatch

## Abstract

DNA fingerprinting data for 4,207 *Mycobacterium tuberculosis* isolates were combined with data from a computer program (Origins). Largest population groups were from England (n = 1,031) and India (n = 912), and most prevalent strains were the Euro-American (45%) and East African–Indian (34%) lineages. Combining geographic and molecular data can enhance cluster investigation.

Knowledge and understanding of transmission dynamics of *Mycobacterium tuberculosis* have been improved by development of rapid molecular techniques that are being more extensively applied (*1**–**3*). Globally, application of molecular techniques has identified major *M*. *tuberculosis* lineages associated with geographic origin (*4**–**7*). Previous studies on transmission dynamics of *M*. *tuberculosis* have usually analyzed patient-declared population groups to identify associations (*1**,**7*).

We describe a novel software (Origins; Experian, Nottingham, UK) that assigns cultural, ethnic, and linguistic (CEL) groups on the basis of given and family names. Records from 12 countries containing 1,600,000 family and 600,000 given names were analyzed to construct >200 origin types based on CEL factors associated with given and family names. This approach is applicable worldwide and is more accurate and has better coverage than other software (*8*). The first use of Origins in healthcare was identification of how a European CEL group came to emergency departments in the United Kingdom (*9*).

The aim of this study was to combine mycobacterial fingerprinting data and patient origin as assigned by Origins to relate the occurrence of major global *M*. *tuberculosis* lineages in populations originating from around the world. Combining data obtained from universal typing and associated cultural and social links identified by Origins provides the potential for a deeper understanding of the causes for distribution of prevalent strains in specific population groups.

## The Study

Nonduplicate initial *M*. *tuberculosis* complex isolates (n = 4,207) were referred from the Midlands region of the United Kingdom (population 9.5 million) to our center during January 2004–December 2007. These isolates were incubated, identified, and analyzed by mycobacterial interspersed repetitive units containing variable numbers of tandem repeats (MIRU-VNTR) typing (*10*). MIRU-VNTR typing analyzes the number of repetitive DNA sequences at multiple independent genetic loci. These data were compared with those in an online database (MIRU-VNTRplus), which was developed by Allix-Beguec et al. (*11*). This database was used to assign *M*. *tuberculosis* strains to 1 of 6 lineages: East African–Indian, East Asian, Euro-American, Indo-Oceanic, West African-1, or West African-2.

The given and family names of 4,207 patients were entered into Origins to obtain a CEL group for each, which was then assigned a continent on the basis of the United Nations Standard Country and Area Codes Classification Scheme (*12*). Origins can assign a CEL group when the given and family names are present in a dataset.

Within the study population are predominant CEL groups that originate from each continent: 1,031 (25%) from England in Europe, 912 (22%) from India in Asia, and 130 (3%) from Somalia in Africa (Table 1). The 18 isolates from the Americas represented 3 CEL groups. Origins assigned 4,175 (99%) of 4,207 patients to 77 CEL groups; 32 patients were unclassified.

**Table 1:** *Mycobacterium tuberculosis* isolates from CEL groups, the Midlands, UK*

Continent and CEL group	No. (%) isolates
Africa	263 (6)
Somalia	130 (3)
Other, n = 18 groups	133 (3)
North and South America	
CEL group, n = 3 groups	18 (0)
Asia	2,421 (58)
India	912 (22)
Pakistan	777 (18)
Pakistan–Kashmir	212 (5)
Bangladesh	199 (5)
Northern India	95 (2)
Other, n = 22 groups	226 (5)
Europe	1,473 (35)
England	1,031 (25)
Ireland	123 (3)
Scotland	99 (2)
Wales	98 (2)
Other, n = 23 groups	122 (3)
Unclassified	32 (0)
Total	4,207 (100)

*CEL, cultural, ethnic, and linguistic. CEL groups representing <1% (42 isolates) of the total are not shown.

Using the 15 MIRU-VNTR loci, we matched 4,117 (98%) of 4,207 typed strains to strains in the MIRU-VNTRplus database. The 90 strains that did not match with 1 of the 6 major global lineages were *M*. *bovis* (24 strains) or could not be definitively assigned (66 strains) to 1 of the 6 global lineages. Continental and regional origins of patients as assigned by Origins and global lineage were then combined to identify the distribution of global *M*. *tuberculosis* lineages within each population (Table 2).

**Table 2:** Distribution of *Mycobacterium tuberculosis* isolates according to lineage and continent of patient origin on the basis of CEL group, the Midlands, UK*

Continent and region	No. (%) isolates in each *M. tuberculosis* lineage						Total no. (%) isolates
	East African–Indian	East Asian	Euro-American	Indo-Oceanic	West African-1	West African-2	
Africa							
Eastern	56 (4)	9 (4)	62 (3)	39 (7)	0	0	166 (4)
Central	0	0	4 (0)	1 (0)	0	0	5 (0)
Northern	2 (0)	0	3 (0)	1 (0)	0	0	6 (0)
Southern	2 (0)	1 (0)	29 (2)	3 (1)	0	0	35 (1)
Western	5 (0)	3 (1)	21 (1)	5 (1)	4 (24)	1 (14)	39 (1)
Unknown	0	1 (0)	6 (0)	1 (0)	0	0	8 (0)
Region total	65 (5)	14 (6)	125 (7)	50 (9)	4 (24)	1 (14)	259 (6)
Americas							
Caribbean region	2 (0)	0	5 (0)	0	0	0	7 (0)
North America	2 (0)	0	4 (0)	1	0	0	7 (0)
South America	2 (0)	0	2 (0)	0	0	0	4 (0)
Region total	6 (0)	0	11 (1)	1 (0)	0	0	18 (0)
Asia							
Eastern	4 (0)	25 (11)	10 (1)	5 (1)	0	0	44 (1)
Southeastern	1 (0)	2 (1)	3 (0)	5 (1)	0	0	11 (0)
Southern	1,117 (79)	100 (46)	614 (32)	403 (72)	4 (24)	2 (29)	2,240 (55)
Western	5 (0)	0	13 (1)	1 (0)	0	0	19 (0)
Unknown	23 (2)	1 (0)	23 (1)	5 (1)	0	0	52 (1)
Region total	1,150 (81)	128 (59)	663 (35)	419 (75)	4 (24)	2 (29)	2,366 (58)
Europe							
Eastern	1 (0)	1 (0)	17 (1)	2 (0)	0	0	21 (1)
Northern	186 (13)	69 (32)	994 (52)	74 (13)	7 (41)	4 (57)	1,334 (33)
Southern	9 (1)	6 (3)	40 (2)	6 (1)	1 (6)	0	62 (2)
Western	0	0	14 (1)	0	1 (6)	0	15 (0)
Unknown	1 (0)	0	7 (0)	2 (0)	0	0	10 (0)
Region total	197 (14)	76 (35)	1,072 (57)	84 (15)	9 (53)	4 (57)	1,442 (35)
Unclassified	7 (0)	0	23 (1)	2 (0)	0	0	32 (1)
Total	1,425 (100)	218 (100)	1,894 (100)	556 (100)	17 (100)	7 (100)	4,117 (100)

*CEL, cultural, ethnic, and linguistic. Unknown indicates that the continent was identified but without a specific region. The United Kingdom (Great Britain and Northern Ireland) is located in northern Europe and India, Pakistan, and Bangladesh are located in southern Asia. A total of 90 (2%) of 4,207 strains were not assigned to 1 of the 6 major lineages.

The Euro-American lineage was the most prevalent lineage in our study. It contained 1,894 (45%) strains and was present in each continental human population group. The Euro-American strain was the most prevalent lineage in patients originating from Africa (125), the Americas (11), and Europe (1,072) and was the second most prevalent lineage in patients originating from Asia (663). The most prevalent *M*. *tuberculosis* lineage in patients originating from Asia was the East African–Indian lineage (1,150).

Combining geographic data assigned by Origins and DNA fingerprinting data could affect public health efforts to control tuberculosis because this approach can identify strains in CEL groups in which specific global *M*. *tuberculosis* lineages are not present. The MIRU-VNTR profile 424352332515333 (East Asian lineage) was identified in 23 patients from the Midlands. Of these 23 patients, 20 resided within a 5-mile radius of each other. Within this geographically restricted cluster, 12 (60%) of these patients were assigned to the Europe CEL group and 8 patients to part of the Asia CEL group. The first strain was identified in 2004, and subsequent strains were identified in each year of this study.

The MIRU-VNTR profile 422352542517333 was identified in 102 patients during 2004–2007. This profile was matched with the East African–Indian lineage; 98 (96%) patients originated from Asia and 4 (4%) from Europe. This strain was identified in various locations in the Midlands within an ≈40-mile radius that included all patients.

## Conclusions

We studied >4,000 *M*. *tuberculosis* isolates typed in the United Kingdom. Our study demonstrated that the combination of molecular and population group data provided by novel software can provide information about the molecular epidemiology of *M*. *tuberculosis*.

The 2 example MIRU-VNTR profiles show that molecular and social data identified an East Asian strain in an unsuspected CEL group (Europe) and limited transmission of an East African–Indian strain between CEL groups. Geographic restriction of the 424352332515333 East Asian strain in the European CEL group identified possible recent transmission within this population group. The 422352542517333 East African–Indian strain infected a large number of patients (102) and showed wide geographic spread with limited transmission into the European CEL group (4/102 patients). This finding indicates that this strain is widely distributed in southern Asia and has not been transmitted between CEL groups. Its wide distribution in the United Kingdom reflects areas of residence for this CEL group.

Data from our study support previous findings and extend the dataset for Europe. Our results also include a large number of strains from southern Asia, which were underrepresented in other studies (*7**,**13*).

Origins identified CEL groups within a country (e.g., Kashmir in Pakistan or northern India) and divided Great Britain and Ireland into 4 CEL groups (Table 1). This enhanced differentiation could be useful in future population-based studies because migration patterns may be localized to specific areas within countries and common social networks could be identified. CEL groups can be assigned to any dataset in which the patient’s name is known. Traditional epidemiologic identification of ethnic groups requires a questionnaire, but if patient names are not in a dataset, then CEL groups cannot be assigned. Origins showed some discrepancies because the black Caribbean CEL group usually has British names and will be assigned as a British CEL group (*8*). However, the utility of Origins is maximized when it is applied to diverse populations.

Many countries now routinely type *M*. *tuberculosis* isolates by using MIRU-VNTR typing. This analysis identifies clusters of strain types across place and time. By using Origin software for identification of CEL groups, public health officials can identify and investigate possible cultural links for transmission of *M*. *tuberculosis*.
